# Supplementary material for: Systematic Review and Network Meta-Analysis of Immune Checkpoint Inhibitors in Combination with Chemotherapy as a First-Line Therapy for Extensive-Stage Small Cell Carcinoma
Source: Cancers (Basel). 2020 Dec 3;12(12):3629. doi: 10.3390/cancers12123629 (PMC7761843; doi:10.3390/cancers12123629)
Supplement: Supplementary file 1 [file cancers-12-03629-s001.pdf]

Supplementary Materials: Systematic Review and Network Meta-Analysis of Immune Checkpoint Inhibitors in Combination with Chemotherapy as a First-Line Therapy for Extensive-Stage Small Cell Carcinoma

Table S1. List of Excluded Papers with Reasons.

List of Excluded Papers with Reasons
Included limited stage SCLC (*n* = 1)
Hellmann, M.D.; Callahan, M.K.; Awad, M.M.; Calvo, E.; Ascierto, P.A.; Atmaca, A.; Rizvi, N.A.; Hirsch, F.R.; Selvaggi, G.; Szustakowski, J.D.; et al. Tumor Mutational Burden and Efficacy of Nivolumab Monotherapy and in Combination with Ipilimumab in Small-Cell Lung Cancer. Cancer Cell 2018, 33, 853–861.e4, doi:10.1016/j.ccell.2018.04.001
Non-randomized RCTs (*n* = 7)
Hellmann, M.D.; Callahan, M.K.; Awad, M.M.; Calvo, E.; Ascierto, P.A.; Atmaca, A.; Rizvi, N.A.; Hirsch, F.R.; Selvaggi, G.; Szustakowski, J.D.; et al. Tumor Mutational Burden and Efficacy of Nivolumab Monotherapy and in Combination with Ipilimumab in Small-Cell Lung Cancer. Cancer Cell 2018, 33, 853–861.e4, doi:10.1016/j.ccell.2018.04.001. [Google Search] [Google Scholar] [Google Books] [Publisher website]
Gelsomino, F.; Lamberti, G.; Parisi, C.; Casolari, L.; Melotti, B.; Sperandi, F.; Ardizzoni, A. The evolving landscape of immunotherapy in small-cell lung cancer: A focus on predictive biomarkers. *Cancer Treat. Rev.* **2019**, *79*, 101887, doi:10.1016/j.ctrv.2019.08.003.
Reck, M.; Heigener, D.; Reinmuth, N. Immunotherapy for small-cell lung cancer: emerging evidence. *Futur. Oncol.* **2016**, *12*, 931–943, doi:10.2217/fon-2015-0012.
Pacheco, J.M.; Bunn, P.A. Advancements in Small-cell Lung Cancer: The Changing Landscape Following IMpower-133. *Clin. Lung Cancer* **2019**, *20*, 148–160.e2, doi:10.1016/j.cllc.2018.12.019.
Krug, L.M.; Milton, D.; Chen, L.; Jungbluth, A.A.; Quaia, E.; Nagel, A.; Jones, J.; Finn, R.; Divgi, C. Targeting Lewis Y (LeY) in small cell lung cancer (SCLC) with a humanized monoclonal antibody, hu3S193. *J. Clin. Oncol.* **2006**, *24*, 7086, doi:10.1200/jco.2006.24.18_suppl.7086.
Ribas, A. Genetically modified dendritic cells for cancer immunotherapy. *Curr. Gene Ther.* **2005**, *5*, 619–28, doi:10.2174/156652305774964758.
Zhang, X.; Li, F.; Sun, S.-J.; Hu, Y.; Wang, G.; Wang, Y.; Cui, X.-X.; Jiao, S. Adoptive Immunotherapy for Small Cell Lung Cancer by Expanded Activated Autologous Lymphocytes: a Retrospective Clinical Analysis. *Asian Pac. J. Cancer Prev.* **2015**, *16*, 1487–1494, doi:10.7314/apjcp.2015.16.4.1487.
No comparison group (*n* = 2)
Welsh, J.W.; Heymach, J.V.; Chen, D.; Verma, V.; Cushman, T.R.; Hess, K.R.; Shroff, G.; Tang, C.; Skoulidis, F.; Jeter, M.; et al. Phase I Trial of Pembrolizumab and Radiation Therapy after Induction Chemotherapy for Extensive-Stage Small Cell Lung Cancer. *J. Thorac. Oncol.* **2020**, *15*, 266–273, doi:10.1016/j.jtho.2019.10.001.
Levy, A.; Massard, C.; Soria, J.-C.; Deutsch, E. Concurrent irradiation with the anti-programmed cell death ligand-1 immune checkpoint blocker durvalumab: single centre subset analysis from a phase 1/2 trial. *Eur. J. Cancer*, **2016**, *68*, 156-162.
Cost effectiveness analysis (*n* = 2)
Li, L.-Y.; Wang, H.; Chen, L.-M.; Li, W.-Q.; Cui, J.-W. First-line atezolizumab plus chemotherapy in treatment of extensive small cell lung cancer. *Chin. Med J.* **2019**, *132*, 2790–2794, doi:10.1097/cm9.0000000000000536..
Zhou, K.; Zhou, J.; Huang, J.; Zhang, N.; Bai, L.; Yang, Y.; Li, Q. Cost-effectiveness analysis of atezolizumab plus chemotherapy in the first-line treatment of extensive-stage small-cell lung cancer. *Lung Cancer* **2019**, *130*, 1–4, doi:10.1016/j.lungcan.2019.01.019.
Subgroup analysis of included RCTs (*n* = 1).
Nishio, M.; Sugawara, S.; Atagi, S.; Akamatsu, H.; Sakai, H.; Okamoto, I.; Takayama, K.; Hayashi, H.; Nakagawa, Y.; Kawakami, T. Subgroup Analysis of Japanese Patients in a Phase III Study of Atezolizumab in Extensive-stage Small-cell Lung Cancer (IMpower133). *Clin. Lung Cancer* **2019**, *20*, 469–476.e1, doi:10.1016/j.cllc.2019.07.005.
Treatment protocol was different (*n* = 2)
Reck, M.; Bondarenko, I.; Luft, A.; Serwatowski, P.; Barlesi, F.; Chacko, R.; Sebastian, M.; Lu, H.; Cuillerot, J.-M.; Lynch, T.J. Ipilimumab in combination with paclitaxel and carboplatin as first-line therapy in extensive-disease-small-cell lung cancer: results from a randomized, double-blind, multicenter phase 2 trial. *Ann. Oncol.* **2013**, *24*, 75–83, doi:10.1093/annonc/mds213.
Von Pawel, J.; Kim, S.-W.; Spigel, D.R.; Zielinski, C.; Pietanza, M.C.; De Pril, V.; Ballas, M.S.; Reck, M. CA184-156: Randomized, multicenter, double-blind, phase III trial comparing the efficacy of ipilimumab (Ipi) plus etoposide/platinum (EP) versus placebo plus EP in patients (Pts) with newly diagnosed extensive-stage disease small cell lung cancer (ED-SCLC). *J. Clin. Oncol.* **2013**, *31*, TPS7608, doi:10.1200/jco.2013.31.15_suppl.tps7608.

Patient-reported outcomes (n = 2)
Mansfield, A.S.; Każarnowicz, A.; Karaseva, N.; Sánchez, A.; De Boer, R.; Andric, Z.; Reck, M.; Atagi, S.; Lee, J.-S.; Garassino, M.; et al. Safety and patient-reported outcomes of atezolizumab, carboplatin, and etoposide in extensive-stage small-cell lung cancer (IMpower133): a randomized phase I/III trial. *Ann. Oncol.* **2020**, *31*, 310–317, doi:10.1016/j.annonc.2019.10.021.
Goldman, J.W.; Garassino, M.C.; Chen, Y.; Özgüroğlu, M.; Dvorkin, M.; Trukhin, D.; Statsenko, G.; Hotta, K.; Ji, J.H.; Hochmair, M.J.; et al. Patient-reported outcomes with first-line durvalumab plus platinum-etoposide versus platinum-etoposide in extensive-stage small-cell lung cancer (CASPIAN): a randomized, controlled, open-label, phase III study. *Lung Cancer* **2020**, doi:10.1016/j.lungcan.2020.09.003.
Not focused on newly diagnosed ESCLC without prior chemotherapy (n = 7)
Pujol, J.-L.; Greillier, L.; Audigier-Valette, C.; Moro-Sibilot, D.; Uwer, L.; Hureaux, J.; Guisier, F.; Carmier, D.; Madelaine, J.; Otto, J.; et al. A Randomized Non-Comparative Phase II Study of Anti-Programmed Cell Death-Ligand 1 Atezolizumab or Chemotherapy as Second-Line Therapy in Patients With Small Cell Lung Cancer: Results From the IFCT-1603 Trial. *J. Thorac. Oncol.* **2019**, *14*, 903–913, doi:10.1016/j.jtho.2019.01.008.
Cope, S.; Keeping, S.T.; Goldgrub, R.; Ayers, D.; Jansen, J.P.; Penrod, J.R.; Korytowsky, B.; Juarez-Garcia, A.; Yuan, Y. Indirect comparison of nivolumab ± ipilimumab (CheckMate 032) versus other treatments for recurrent small-cell lung cancer. *J. Comp. Eff. Res.* **2019**, *8*, 733–751, doi:10.2217/cer-2018-0130.
Chiappori, A.A.; Williams, C.C.; Gray, J.E.; Tanvetyanon, T.; Haura, E.B.; Creelan, B.C.; Thapa, R.; Chen, D.-T.; Simon, G.R.; Bepler, G.; et al. Randomized-controlled phase II trial of salvage chemotherapy after immunization with a TP53-transfected dendritic cell-based vaccine (Ad.p53-DC) in patients with recurrent small cell lung cancer. *Cancer Immunol. Immunother.* **2018**, *68*, 517–527, doi:10.1007/s00262-018-2287-9.
Roszkowski, K.; Nozdryn-Plotnicki, B.; Roszkowski, W.; Ko, H.L.; Jeljaszewicz, J.; Pulverer, G. Small-cell lung cancer and immunochemotherapy with Propionibacterium granulosum KP 45. *J Cancer Res Clin Oncol* 1985, *109*, 72–77.
Navarro, A.; Felip, E. Pembrolizumab in advanced pretreated small cell lung cancer patients with PD-L1 expression: data from the KEYNOTE-028 trial: a reason for hope? *Transl. Lung Cancer Res.* **2017**, *6*, S78–S83, doi:10.21037/tlcr.2017.10.04.
Zarogoulidis, K.; Ziogas, E.; Boutsikou, E.; Zarogoulidis, P.; Darwiche, K.; Kontakiotis, T.; Tsakiridis, K.; Porpodis, K.; Latsios, D.; Chatzizisi, O.; et al. Immunomodifiers in combination with conventional chemotherapy in small cell lung cancer: a Phase II, randomized study. *Drug Des. Dev. Ther.* **2013**, *7*, 611–617, doi:10.2147/DDDT.S43184.
Arnold, S.M.; Chansky, K.; Baggstrom, M.Q.; Thompson, M.A.; Sanborn, R.E.; Villano, J.L.; Waqar, S.N.; Hamm, J.; Leggas, M.; Willis, M.; et al. Phase II Trial of Carfilzomib Plus Irinotecan in Patients With Small-cell Lung Cancer Who Have Progressed on Prior Platinum-based Chemotherapy. *Clin. Lung Cancer* **2020**, *21*, 357–364.e7, doi:10.1016/j.cllc.2020.01.006.
Meta-analysis (n = 2)
Facchinetti, F.; Di Maio, M.; Tiseo, M. Adding PD-1/PD-L1 Inhibitors to Chemotherapy for the First-Line Treatment of Extensive Stage Small Cell Lung Cancer (SCLC): A Meta-Analysis of Randomized Trials. *Cancers* **2020**, *12*, 2645, doi:10.3390/cancers12092645.
Landre, T.; Chouahnia, A.; Guetz, G.D.; Assié, J.-B.; Chouaid, C. 1799P Immune checkpoint inhibitor plus chemotherapy versus chemotherapy alone as first-line for extensive stage small cell lung cancer: A meta-analysis. *Ann. Oncol.* **2020**, *31*, S1041, doi:10.1016/j.annonc.2020.08.1560.
Duplicate RCT (were published in different journals or at a conference) (n = 4)
Goldman J.W., Garassino M.C., Chen Y. et al. Durvalumab (D) ± tremelimumab (T) + platinum-etoposide (EP) in 1L ES-SCLC: Characterization of long-term clinical benefit and tumour mutational burden (TMB) in CASPIAN. Annals of Oncology 2020 31 Supplement 4 (S1212-S1213).
Liu, S.; Horn, L.; Mok, T.; Mansfield, A.; De Boer, R.; Losonczy, G.; Sugawara, S.; Dziadziuszko, R.; Krzakowski, M.; Smolin, A.; et al. 1781MO IMpower133: Characterisation of long-term survivors treated first-line with chemotherapy ± atezolizumab in extensive-stage small cell lung cancer. *Ann. Oncol.* **2020**, *31*, S1032–S1033, doi:10.1016/j.annonc.2020.08.1543.
Reck, M.; Liu, S.; Mansfield, A.; Mok, T.; Scherpereel, A.; Reinmuth, N.; Garassino, M.; De Carpeno, J.; Califano, R.; Nishio, M.; et al. IMpower133: Updated overall survival (OS) analysis of first-line (1L) atezolizumab (atezo) + carboplatin + etoposide in extensive-stage SCLC (ES-SCLC). *Ann. Oncol.* **2019**, *30*, v710–v711, doi:10.1093/annonc/mdz264.
Rudin, C.; Shen, L.; Pietanza, M. P2.04-007 KEYNOTE-604: Phase 3 Randomized, Double-Blind Trial of Pembrolizumab/Placebo plus Etoposide/Platinum for Extensive Stage-SCLC. *J. Thorac. Oncol.* **2017**, *12*, S2400, doi:10.1016/j.jtho.2017.11.020.
Protocol (n = 1)
Ramirez, R.A.; Matrana, M.R.; Satti, S.; Griffin, R.P.; A Voros, B.; Bren-Mattison, Y. A phase II trial of pembrolizumab in combination with cisplatin or carboplatin and etoposide in chemotherapy naive patients with metastatic or unresectable high-grade gastroenteropanncreatic or lung neuroendocrine carcinoma. *J. Clin. Oncol.* **2020**, *38*, TPS635, doi:10.1200/jco.2020.38.4_suppl.tps635.

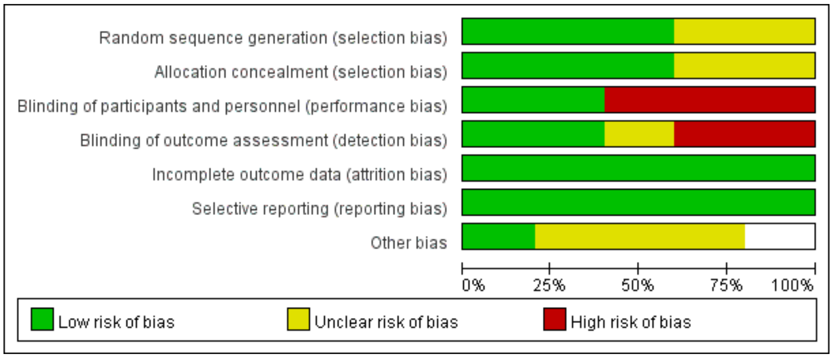

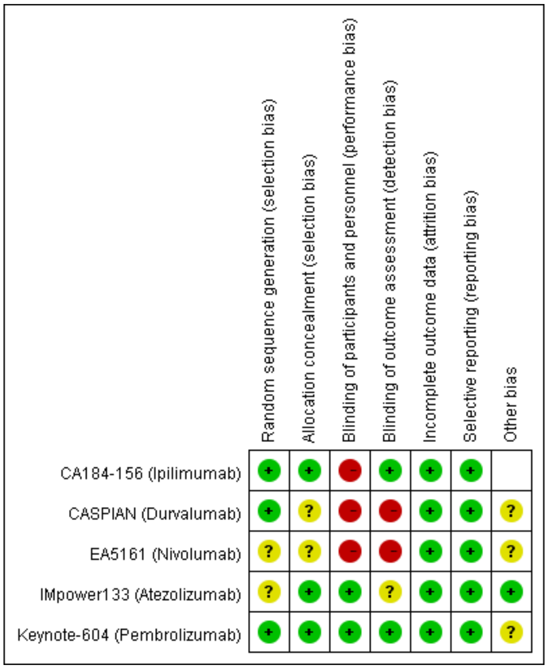

Figure S1. Quality Assessment Using the Risk of Bias Tool (ROB Tool). Bias Tool which was performed using Review Manager version 5.1.

A.

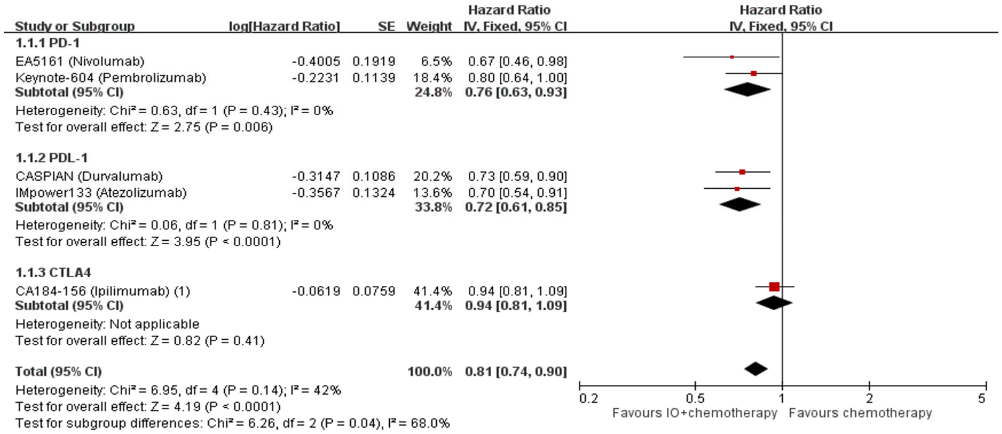

B.

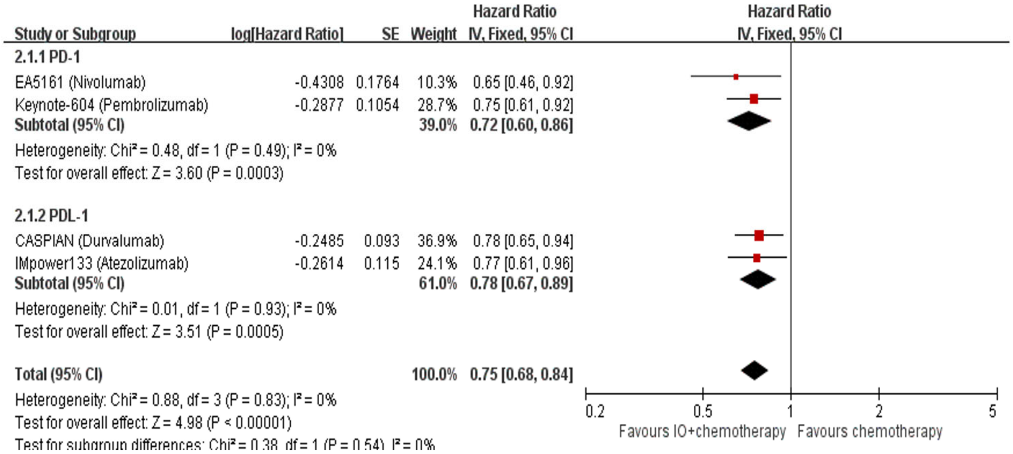

C.

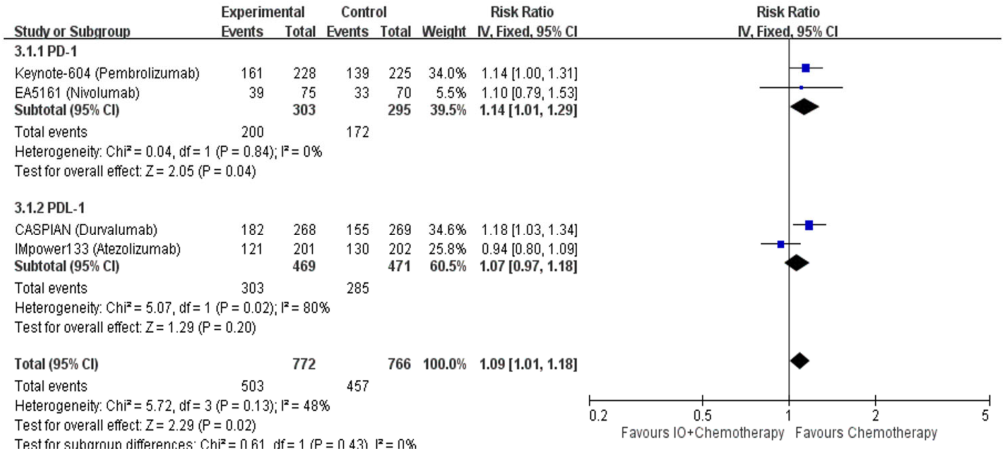

Figure S2. Forest Plot and Pooled HR 95% CI for (**A**) Overall survival; (**B**) Progression free survival; (**C**) Objective response rate.

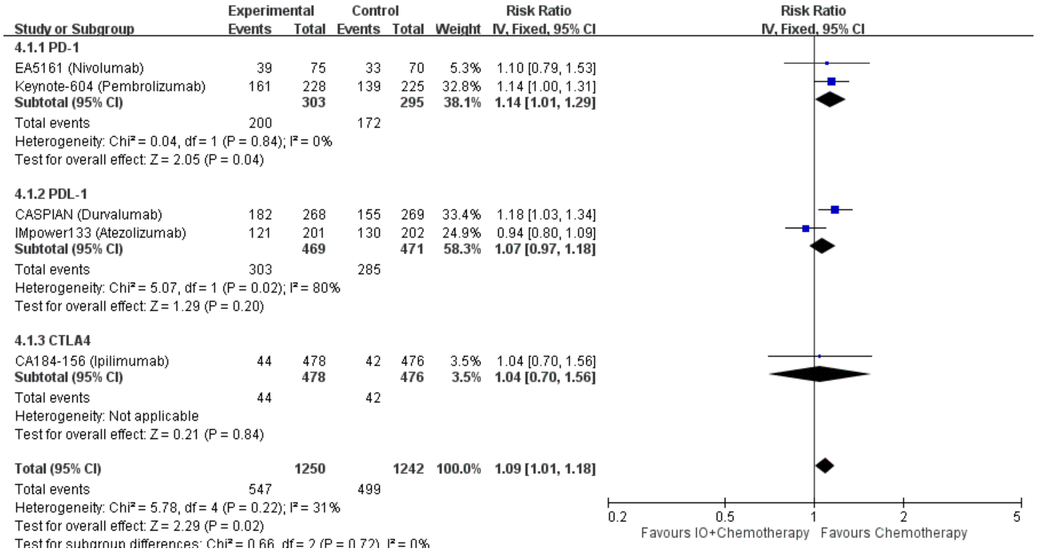

Figure S3. Forest Plot and Pooled HR 95% CI for Grade 3–4 Adverse Events.

A.

CTX (SUCRA=19.4%, OS rank=4)				
0.93 (0.78,1.12)	Ipili+CTX (SUCRA=7.8%, OS rank=5)			
1.47 (1.15,1.89)	1.57 (1.15,2.15)	Pembro+CTX (SUCRA=83.7%, OS rank=1)		
1.32 (1.02,1.69)	1.41 (1.03,1.93)	0.89 (0.63,1.28)	Durva+CTX (SUCRA=67.7%, OS rank=3)	
1.35 (0.99,1.85)	1.45 (1.00,2.08)	0.92 (0.61,1.37)	1.03 (0.69,1.54)	Atezo+CTX (SUCRA=71.4%, OS rank=2)

B.

CTX (SUCRA=19.0%, OS rank=4)				
0.94 (0.72,1.23)	Ipili+CTX (SUCRA=12.5%, OS rank=5)			
1.28 (0.92,1.79)	1.36 (0.89,2.08)	Pembro+CTX (SUCRA=57.1%, OS rank=3)		
1.59 (1.01,2.50)	1.68 (0.99,2.85)	1.24 (0.71,2.17)	Durva+CTX (SUCRA=81.8%, OS rank=1)	
1.54 (0.99,2.38)	1.63 (0.98,2.72)	1.20 (0.69,2.08)	0.97 (0.52,1.82)	Atezo+CTX (SUCRA=79.6%, OS rank=2)

C.

CTX (SUCRA=28.3%, OS rank=4)				
0.93 (0.77,1.11)	Ipili+CTX (SUCRA=12.3%, OS rank=5)			
1.32 (0.99,1.75)	1.42 (1.01,2.00)	Pembro+CTX (SUCRA=78.8%, OS rank=2)		
1.35 (1.02,1.79)	1.46 (1.05,2.04)	1.03 (0.69,1.53)	Durva+CTX (SUCRA=84.3%, OS rank=1)	
1.09 (0.76,1.56)	1.17 (0.78,1.76)	0.83 (0.52,1.31)	0.80 (0.51,1.27)	Atezo+CTX (SUCRA=46.3%, OS rank=3)

D.

CTX (SUCRA=1.7%, OS rank=4)			
1.43 (1.08,1.89)	Pembro+CTX (SUCRA=57.5%, OS rank=2)		
1.33 (0.96,1.85)	0.93 (0.61,1.44)	Durva+CTX (SUCRA=47.6%, OS rank=3)	
1.89 (1.28,2.78)	1.32 (0.82,2.13)	1.42 (0.85,2.35)	Atezo+CTX (SUCRA=93.2%, OS rank=1)

E.

CTX (SUCRA=47.7%, OS rank=3)				
0.63 (0.41,0.98)	Ipili+CTX (SUCRA=7.2%, OS rank=5)			
1.11 (0.83,1.49)	1.76 (1.04,2.98)	Pembro+CTX (SUCRA=64.7%, OS rank=2)		
1.45 (0.74,2.86)	2.29 (1.02,5.13)	1.30 (0.62,2.73)	Durva+CTX (SUCRA=85.3%, OS rank=1)	
0.93 (0.41,2.13)	1.48 (0.58,3.75)	0.84 (0.35,2.02)	0.64 (0.22,1.87)	Atezo+CTX (SUCRA=45.1%, OS rank=4)

F.

CTX (SUCRA=15.8%, OS rank=4)				
0.97 (0.83,1.14)	Ipili+CTX (SUCRA=9.8%, OS rank=5)			
1.45 (1.16,1.82)	1.49 (1.13,1.97)	Pembro+CTX (SUCRA=76.9%, OS rank=2)		
1.35 (1.08,1.69)	1.39 (1.06,1.83)	0.93 (0.68,1.28)	Durva+CTX (SUCRA=66.5%, OS rank=3)	
1.47 (1.12,1.92)	1.51 (1.11,2.07)	1.01 (0.71,1.44)	1.09 (0.77,1.55)	Atezo+CTX (SUCRA=81.0%, OS rank=1)

G.

CTX (SUCRA=28.9%, OS rank=4)				
0.78 (0.60,1.02)	Ipili+CTX (SUCRA=2.3%, OS rank=5)			
1.67 (1.11,2.50)	2.13 (1.31,3.47)	Pembro+CTX (SUCRA=87.0%, OS		
1.41 (0.95,2.08)	1.80 (1.12,2.90)	0.85 (0.48,1.48)	Durva+CTX (SUCRA=72.2%, OS	
1.27 (0.79,2.04)	1.62 (0.94,2.80)	0.76 (0.41,1.42)	0.90 (0.48,1.67)	Atezo+CTX (SUCRA=59.6%, OS rank=3)

H.

CTX (SUCRA=12.2%, OS rank=5)				
1.01 (0.85,1.20)	Ipili+CTX (SUCRA=17.2%, OS rank=4)			
1.27 (1.01,1.59)	1.25 (0.94,1.67)	Pembro+CTX (SUCRA=62.8%, OS		
1.32 (1.02,1.69)	1.30 (0.96,1.77)	1.04 (0.74,1.46)	Durva+CTX (SUCRA=70.4%, OS	
1.47 (1.08,2.00)	1.46 (1.02,2.08)	1.16 (0.79,1.70)	1.12 (0.75,1.66)	Atezo+CTX (SUCRA=87.4%, OS rank=1)

Figure S4. League Table with NMA Estimates for Subgroup Analysis of Overall Survival. (**A**) Male; (**B**) Female; (**C**) Age < 65; (**D**) Age ≥65; (**E**) Patient with brain metastases; (**F**) Patient without brain metastases; (**G**) Eastern Cooperative Oncology Group (ECOG) = 0; (H)ECOG = 1; * CTX: chemotherapy; Ipili: Ipilimumab; Pembro:Pembrolizumab; Durva: Durvalumab; Atezo: Atezolizumab.

A.

CTX (SUCRA=47.4%, SUCRA rank=4)					
1.17 (0.63,2.17)	Ipili+CTX (SUCRA=34.8%, SUCRA rank=5)				
0.81 (0.49,1.32)	0.69 (0.31,1.52)	Pembro+CTX (SUCRA=72.3%, SUCRA rank=2)			
1.66 (0.90,3.08)	1.42 (0.59,3.39)	2.06 (0.93,4.54)	Durva+CTX (SUCRA=8.8%, SUCRA rank=6)		
0.76 (0.40,1.44)	0.65 (0.27,1.57)	0.94 (0.42,2.11)	0.46 (0.19,1.11)	Atezo+CTX (SUCRA=75.3%, SUCRA rank=1)	
0.89 (0.49,1.62)	0.76 (0.32,1.79)	1.10 (0.51,2.39)	0.54 (0.23,1.26)	1.17 (0.49,2.82)	Nivo+CTX (SUCRA=61.4%, SUCRA rank=3)

B.

CTX (SUCRA=56.6%, SUCRA rank=4)					
1.34 (0.90,1.99)	Ipili+CTX (SUCRA=25.2%, SUCRA rank=5)				
0.97 (0.63,1.50)	0.73 (0.40,1.30)	Pembro+CTX (SUCRA=60.3%, SUCRA rank=3)			
1.99 (1.26,3.15)	1.49 (0.81,2.73)	2.05 (1.09,3.86)	Durva+CTX (SUCRA=2.8%, SUCRA rank=6)		
0.87 (0.52,1.44)	0.65 (0.34,1.23)	0.89 (0.46,1.74)	0.43 (0.22,0.86)	Atezo+CTX (SUCRA=72.0%, SUCRA rank=2)	
0.75 (0.41,1.36)	0.56 (0.27,1.14)	0.77 (0.37,1.61)	0.38 (0.18,0.80)	0.87 (0.40,1.89)	Nivo+CTX (SUCRA=83.0%, SUCRA rank=1)

C.

CTX (SUCRA=72.6%, SUCRA rank=2)					
1.64 (1.25,2.16)	Ipili+CTX (SUCRA=4.4%, SUCRA rank=6)				
0.94 (0.75,1.17)	0.57 (0.40,0.81)	Pembro+CTX (SUCRA=84.2%, SUCRA rank=1)			
1.37 (1.04,1.80)	0.83 (0.57,1.22)	1.46 (1.03,2.07)	Durva+CTX (SUCRA=21.6%, SUCRA rank=5)		
1.08 (0.76,1.54)	0.66 (0.42,1.02)	1.15 (0.76,1.74)	0.79 (0.50,1.23)	Atezo+CTX (SUCRA=57.6%,SUCRA rank=4)	
1.06 (0.83,1.37)	0.65 (0.45,0.94)	1.13 (0.81,1.58)	0.78 (0.54,1.12)	0.99 (0.64,1.52)	Nivo+CTX (SUCRA=59.5%,SUCRA rank=3)

Figure S5. League Table with NMA Estimates for Specific Adverse Effects. (**A**)Thrombocytopenia; (**B**)Anemia; (**C**) Neutropenia; * CTX: chemotherapy; Ipili: Ipilimumab; Pembro: Pembrolizumab; Durva: Durvalumab; Atezo: Atezolizumab.

Table S2. Summary of Death Events for the Included RCTs.

Trial Name	Experimental Arm				Control Arm			
	Regimen	Patient Number	Death Number	Death (%)	Regimen	Patient Number	Death Number	Death (%)
PD-1 inhibitors								
Keynote-604	pembrolizumab + EP	223	14	6.28%	EP alone	223	12	5.38%
EA5161	nivolumab + EP	80	9	11.25%	EP alone	80	7	8.75%
PD-L1 inhibitors								
IMpower133	atezolizumab + EP	198	3	1.52%	EP alone	196	3	1.53%
CASPIAN	durvalumab + EP	265	13	4.91%	EP alone	265	15	5.66%
CTLA4 inhibitors								
CA184-156	ipilimumab + EP	478	4	0.84%	EP alone	476	3	0.63%

EP: platinum chemotherapy (carboplatin or cisplatin) with etoposide.

Table S3. Search Strategy in PubMed.

Search Strategy in PubMed		
**Search**	**Query**	**Results**
#1	small cell lung cancer [MeSH Terms] OR "bronchial small cell carcinoma" OR "lung small cell cancer" OR "lung small cell carcinoma" OR "pulmonary small cell carcinoma" OR "small cell bronchial cancer" OR "small cell bronchial carcinoma" OR "small cell lung cancer" OR "small cell lung carcinoma" OR "small cell pulmonary cancer" OR "small cell pulmonary carcinoma"	74,474
#2	((("small cell lung cancer"[Title/Abstract]) OR ("small cell lung carcinoma"[Title/Abstract]))) OR ("SCLC"[Title/Abstract])	73,884
#3	#1 OR #2	75,260

#4	(((“non small cell lung carcinoma”[Title/Abstract]) OR (“non small cell lung neoplasm”[Title/Abstract])) OR (“non small cell lung cancer”[Title/Abstract])) OR (non small cell lung cancer[MeSH Terms])	74,961
#5	#3 NOT #4	12,838
#6	“limited stage”	1362
#7	#5 NOT #6	12,069
#8	immunotherapy[Title/Abstract] OR “brm therapy”[Title/Abstract] OR “biologic response modifier therapy”[Title/Abstract] OR “biological response modifier therapy”[Title/Abstract] OR “immune therapy”[Title/Abstract] OR “immunogenic therapy”[Title/Abstract]	83,173
#9	pembrolizumab[Title/Abstract] OR keytruda[Title/Abstract] OR atezolizumab[Title/Abstract] OR tecentriq[Title/Abstract] OR mpdl3280a[Title/Abstract] OR nivolumab[Title/Abstract] OR opdivo[Title/Abstract] OR durvalumab[Title/Abstract] OR imfinzi[Title/Abstract] OR avelumab[Title/Abstract] OR bavencio[Title/Abstract] OR msb0010718c[Title/Abstract] OR lambrolizumab[Title/Abstract] OR pidilizumab[Title/Abstract] OR ipilimumab[Title/Abstract] OR yervoy[Title/Abstract] OR tremelimumab[Title/Abstract]	9736
#10	#8 OR #9	89,094
#11	(randomized controlled trial[pt] OR controlled clinical trial[pt] OR randomized[tiab] OR placebo[tiab] OR clinical trials as topic[mesh:noexp] OR randomly[tiab] OR trial[ti])	1,325,260
#12	#7 AND #10 AND #11	43
#13	(#12 AND humans[mesh:noexp]) OR (#12 NOT animals[mesh:noexp])	43
#14	#13 NOT (“infant”[mesh] OR “child”[mesh] OR “adolescent”[mesh] OR adolescent[Title/Abstract] OR child*[Title/Abstract] OR infant[Title/Abstract] OR newborn[Title/Abstract]) Sort by: Most Recent	41

Table S4. Search Strategy in Embase.

Search Strategy in Embase		
**Search**	**Query**	**Results**
#1	‘small cell lung cancer’/exp OR ‘bronchial small cell cancer’ OR ‘bronchial small cell carcinoma’ OR ‘lung small cell cancer’ OR ‘lung small cell carcinoma’ OR ‘microcellular lung carcinoma’ OR ‘pulmonary small cell cancer’ OR ‘pulmonary small cell carcinoma’ OR ‘small cell bronchial cancer’ OR ‘small cell bronchial carcinoma’ OR ‘small cell cancer, lung’ OR ‘small cell lung cancer’ OR ‘small cell lung carcinoma’ OR ‘small cell pulmonary cancer’ OR ‘small cell pulmonary carcinoma’	159,576
#2	‘small cell lung cancer’:ab,ti OR ‘small cell lung carcinoma’:ab,ti OR ‘small cell lung neoplasm’:ab,ti OR ‘sclc’:ab,ti OR ((‘small cell lung’ NEAR/3 cancer):ab,ti) OR ((‘small cell lung’ NEAR/3 carcinoma):ab,ti) OR ((‘small cell lung’ NEAR/3 neoplasm):ab,ti)	115,377
#3	#1 OR #2	160,777
#4	‘lung non-small cell carcinoma cell line’:ab,ti OR ‘non small cell lung carcinoma’:ab,ti OR ‘non small cell lung neoplasm’:ab,ti OR ‘nsclc’:ab,ti OR ((non NEAR/2 ‘small cell lung’):ab,ti)	115,833
#5	‘non small cell lung cancer’/exp	159,074
#6	#4 OR #5	173,785
#7	#3 NOT #6	21,705
#8	(limited NEAR/2 stage):ab,ti	2964
#9	#7 NOT #8	20,376
#10	immunotherapy:ab,ti OR ‘brm therapy’:ab,ti OR ‘biologic response modifier therapy’:ab,ti OR ‘biological response modifier therapy’:ab,ti OR ‘biolog* response modifier therapy’:ab,ti OR ‘immune therapy’:ab,ti OR ‘immunogenic therapy’:ab,ti OR ((immu* NEAR/3 therapy):ab,ti) OR ((immu* NEAR/3 treatment*):ab,ti) OR ((‘immune checkpoint’ NEAR/3 inhibitor*):ab,ti) OR ((‘pd1’ NEAR/3 inhibitor*):ab,ti) OR ((‘pdl1’ NEAR/3 inhibitor*):ab,ti) OR ((‘pd-1’ NEAR/3 inhibitor*):ab,ti) OR ((‘pd-l1’ NEAR/3 inhibitor*):ab,ti) OR ((‘programmed death’ NEAR/2 ‘1 inhibitor*’):ab,ti) OR ((‘programmed death ligand’ NEAR/2 ‘1 inhibitor*’):ab,ti)	233,150
#11	pembrolizumab:ab,ti OR keytruda:ab,ti OR ((mk NEAR/2 3475):ab,ti) OR atezolizumab:ab,ti OR tecentriq:ab,ti OR mpdl3280a:ab,ti OR nivolumab:ab,ti OR opdivo:ab,ti OR ((mdx NEAR/2 1106):ab,ti) OR durvalumab:ab,ti OR imfinzi:ab,ti OR ((med NEAR/2 i4736):ab,ti) OR avelumab:ab,ti OR bavencio:ab,ti OR	23,400

	msb0010718c:ab,ti OR lambrolizumab:ab,ti OR pidilizumab:ab,ti OR ((ct NEAR/2 011):ab,ti) OR ipilimumab:ab,ti OR yervoy:ab,ti OR tremelimumab:ab,ti	
#12	#10 OR #11	244,595
#13	‘clinical trial’/de OR ‘randomized controlled trial’/de OR ‘randomization’/de OR ‘single blind procedure’/de OR ‘double blind procedure’/de OR ‘crossover procedure’/de OR (‘randomi?ed controlled’ NEXT/1 trial*) OR rct OR ‘randomly allocated’ OR ‘allocated randomly’ OR ‘random allocation’ OR (allocated NEAR/2 random) OR (single NEXT/1 blind*) OR (double NEXT/1 blind*) OR ((treble OR triple) NEAR/1 blind*)	1,733,540
#14	#9 AND #12 AND #13	194<br>Source Embase = 185<br>PubMed = 8
#15	#14 AND ([young adult]/lim OR [adult]/lim OR [middle aged]/lim OR [aged]/lim OR [very elderly]/lim) AND [humans]/lim	100
